# *ABI3* and *PLCG2* missense variants as risk factors for neurodegenerative diseases in Caucasians and African Americans

**DOI:** 10.1186/s13024-018-0289-x

**Published:** 2018-10-11

**Authors:** Olivia J Conway, Minerva M Carrasquillo, Xue Wang, Jenny M Bredenberg, Joseph S Reddy, Samantha L Strickland, Curtis S Younkin, Jeremy D Burgess, Mariet Allen, Sarah J Lincoln, Thuy Nguyen, Kimberly G Malphrus, Alexandra I Soto, Ronald L Walton, Bradley F Boeve, Ronald C Petersen, John A Lucas, Tanis J Ferman, William P Cheshire, Jay A van Gerpen, Ryan J Uitti, Zbigniew K Wszolek, Owen A Ross, Dennis W Dickson, Neill R Graff-Radford, Nilüfer Ertekin-Taner

**Affiliations:** 1 Department of Neuroscience, Mayo Clinic Florida, Jacksonville, FL 32224 USA; 2 Department of Health Sciences Research, Mayo Clinic Florida, Jacksonville, FL 32224 USA; 3 Department of Neurology, Mayo Clinic Minnesota, Rochester, MN 55905 USA; 4 Department of Psychiatry and Psychology, Mayo Clinic Florida, Jacksonville, FL 32224 USA; 5 Department of Neurology, Mayo Clinic Florida, Jacksonville, FL 32224 USA

**Keywords:** ABI3, PLCG2, Alzheimer’s disease (AD), Progressive supranuclear palsy (PSP), Parkinson’s disease (PD), Dementia with Lewy bodies (DLB), Multiple system atrophy (MSA), African-American, Genetic association, Expression

## Abstract

**Background:**

Rare coding variants *ABI3_*rs616338-T and *PLCG2*_rs72824905-G were identified as risk or protective factors, respectively, for Alzheimer’s disease (AD).

**Methods:**

We tested the association of these variants with five neurodegenerative diseases in Caucasian case-control cohorts: 2742 AD, 231 progressive supranuclear palsy (PSP), 838 Parkinson’s disease (PD), 306 dementia with Lewy bodies (DLB) and 150 multiple system atrophy (MSA) vs. 3351 controls; and in an African-American AD case-control cohort (181 AD, 331 controls). 1479 AD and 1491 controls were non-overlapping with a prior report.

**Results:**

Using Fisher’s exact test, there was significant association of both *ABI3*_rs616338-T (OR = 1.41, *p* = 0.044) and *PLCG2*_rs72824905-G (OR = 0.56, *p* = 0.008) with AD. These OR estimates were maintained in the non-overlapping replication AD-control analysis, albeit at reduced significance (*ABI3*_rs616338-T OR = 1.44, *p* = 0.12; *PLCG2*_rs72824905-G OR = 0.66, *p* = 0.19). None of the other cohorts showed significant associations that were concordant with those for AD, although the DLB cohort had suggestive findings (Fisher’s test: *ABI3*_rs616338-T OR = 1.79, *p* = 0.097; *PLCG2*_rs72824905-G OR = 0.32, *p* = 0.124). *PLCG2*_rs72824905-G showed suggestive association with pathologically-confirmed MSA (OR = 2.39, *p* = 0.050) and PSP (OR = 1.97, *p* = 0.061), although in the opposite direction of that for AD. We assessed RNA sequencing data from 238 temporal cortex (TCX) and 224 cerebellum (CER) samples from AD, PSP and control patients and identified co-expression networks, enriched in microglial genes and immune response GO terms, and which harbor *PLCG2* and/or *ABI3*. These networks had higher expression in AD, but not in PSP TCX, compared to controls. This expression association did not survive adjustment for brain cell type population changes.

**Conclusions:**

We validated the associations previously reported with *ABI3*_rs616338-T and *PLCG2*_rs72824905-G in a Caucasian AD case-control cohort, and observed a similar direction of effect in DLB. Conversely, *PLCG2*_rs72824905-G showed suggestive associations with PSP and MSA in the opposite direction. We identified microglial gene-enriched co-expression networks with significantly higher levels in AD TCX, but not in PSP, a primary tauopathy. This co-expression network association appears to be driven by microglial cell population changes in a brain region affected by AD pathology. Although these findings require replication in larger cohorts, they suggest distinct effects of the microglial genes, *ABI3* and *PLCG2* in neurodegenerative diseases that harbor significant vs. low/no amyloid ß pathology.

**Electronic supplementary material:**

The online version of this article (10.1186/s13024-018-0289-x) contains supplementary material, which is available to authorized users.

## Background

Rare nonsynonymous variants in *ABI3* (p.Ser209Phe; rs616338-T) and *PLCG2* (p.Pro522Arg; rs72824905-G) have recently been implicated in conferring risk and protection, respectively, for Alzheimer’s disease (AD) [1]. Identified using a whole-exome microarray and genotype imputation in the largest AD case-control series to date, these variants are part of a growing number of rare variants now implicated in AD [1]. However, the association of rs616338-T and rs72824905-G has not yet been replicated in an independent cohort and their mechanisms of pathogenesis remain unknown.

*ABI3* encodes the Abelson (Abl) interactor (Abi) family protein 3 (a.k.a. NESH), which is involved in actin cytoskeleton organization and functionally distinct from ABI1 or ABI2 [2]. ABI3 interacts with WASp-family verprolin homologous protein 2 (WAVE2), as part of the WAVE regulatory complex (WRC), a heterocomplex, which also includes NCK-associated proteins (NAP), Specifically Rac-associated 1 (SRA1), and Hematopoietic stem progenitor cell 300 (HSPC300) [3]. WRC activates the actin nucleator actin-related protein-2/3 (Arp2/3) to induce actin polymerization, which is necessary for cell motility in many functions including immune responses [3]. These findings and the identification of ABI3 expressing microglia clusters exclusively in AD brains and around amyloid beta (Aβ) plaques [4] may suggest a role for ABI3 in microglia motility.

*PLCG2* encodes phosphoinositide-specific phospholipase C family protein PLCɣ2, which, upon extracellular ligand stimulation of receptor tyrosine kinase, is activated by recruitment to the cell membrane and phosphorylation [5, 6]. Consequently, PLCɣ2 hydrolyzes phosphatidylinositol 4,5-bisphosphate (PIP2) to inositol 1,4,5-trisphosphate (IP_2_) and diacylglycerol (DAG), which increases calcium (Ca^2+^) influx and extracellular signal-regulated kinase (ERK) phosphorylation, thereby inducing cellular activation in settings including inflammation and innate immunity [5, 6]. The murine *Plcγ2*^Ali5^ gain-of-function point mutation in the catalytic domain of this protein near its auto-inhibitory domain leads to enhanced Ca^2+^ influx in B-cells and expansion of innate inflammatory cells, resulting in autoimmunity and inflammation in this model [7]. Autosomal dominant *PLCG2* in-frame deletion [8] or missense [p.Ser707Tyr] mutations [9] in the autoinhibitory domain, result in constitutively active or hyperactive phospholipase function, respectively, and lead to diseases characterized by autoimmunity and immunodeficiency, known as PLAID (PLCɣ2-associated antibody deficiency) [8] or APLAID (autoinflammation and PLAID) [9].

Given the above and the potential roles of *ABI3* and *PLCG2* in various arms of the immune system, it is likely that the rare missense AD-associated variants within these genes confer their effects through alterations in neuroimmunity, including neuroinflammation, a vital aspect of AD pathophysiology [1, 10]. It is well established that innate and/or adaptive immune changes are observed as features of multiple neurodegenerative diseases as well as in the aging brain [11]. It is therefore possible the *ABI3* and *PLCG2* variants may also influence risk of other neurodegenerative diseases including primary tauopathies and synucleinopathies [11–13]. Identification of association with disease status in other neurodegenerative diseases may aid in understanding the mechanism by which *ABI3* and *PLCG2* contribute to disease pathophysiology. Therefore, in this study, we sought to replicate the association with AD previously observed with *ABI3* rs616338-T and *PLCG2* rs72824905-G in Caucasians, test if this association is also observed in African-Americans, and determine if these variants associate with risk of other neurodegenerative diseases, namely Parkinson’s disease (PD), dementia with Lewy bodies (DLB), progressive supranuclear palsy (PSP) and multiple system atrophy (MSA).

## Methods

### Subjects

A total of 8126 patients were genotyped, of which 7614 were Caucasian (2743 AD, 231 PSP, 855 PD, 306 DLB, 128 MSA, 3351 controls, Table 1) and 512 African American (331 AD, 181 controls). The AD Replication cohort (1480 AD, 1491 controls) is a subset of the AD and control participants in this study that does not overlap with the Mayo Clinic participants included in the Sims et al. [1] publication. All participants were recruited at Mayo Clinic Jacksonville, FL or Mayo Clinic Rochester, MN. Control participants were either cognitively normal at last clinical evaluation or were autopsy controls who had a Braak score of 2.5 or below and without a neuropathological diagnosis of neurodegenerative disease. Of the AD cases 1099 were autopsy confirmed, with a Braak score of 4 or greater, and the remainder had a clinical diagnosis of probable or possible AD [14] by a Mayo Clinic neurologist. All PSP [15] and MSA patients [16] were autopsy confirmed. PD [17] and DLB [18] patients were predominantly clinically diagnosed and of these 77 PD and 67 DLB, patients were pathologically confirmed. Age is defined as age of death for autopsy cases and controls, age at first dementia diagnosis/age at onset for clinically diagnosed cases, and age at last clinical evaluation for cognitively normal controls. The RNA sequencing cohort [19] comprised 86 AD cases, 84 PSP cases and 80 controls, all autopsy confirmed and included in the AD and PSP cohorts.

**Table 1:** Cohort Description

Cohort	N	Mean Age ± SD	Female		APOEε4+	
			N	%	N	%
Caucasian						
Replication AD	1480	80.5 ± 8.9	892	60.27%	831	56.15%
Replication Controls	1491	81.4 ± 8.6	840	56.34%	354	23.74%
AD	2743	79.1 ± 8.5	1648	60.08%	1634	59.57%
PSP	231	74.9 ± 7.2	106	45.89%	42	18.18%
PD	855	62.7 ± 12.0	308	36.02%	248	29.01%
DLB	306	72.9 ± 8.1	69	22.55%	151	49.35%
MSA	128	66.5 ± 8.1	56	43.80%	29	22.66%
Controls	3351	80.6 ± 7.1	1844	55.03%	800	23.87%
African-American						
AD	181	78.8 ± 7.5	130	71.82%	113	62.43%
Controls	331	78.4 ± 7.9	254	76.74%	110	33.23%

Table shows the total number of subjects (N), mean age ± standard deviation (SD), number and percentage of females and the frequency of the APOEε4 allele (%APOEε4+) for subjects retained for analysis. The Replication AD and control cohort is a subset of the AD and control participants in this study non-overlapping with those in Sims et al. [1]

*Abbreviations*: *AD* Alzheimer’s disease, *PSP* progressive supranuclear palsy, *PD* Parkinson’s disease, *DLB* dementia with Lewy bodies, *MSA* multiple system atrophy

### Genotyping

DNA was extracted from blood using AutoGenFlexStar (AutoGen) and FlexiGene Chemistry (Qiagen) or brain using AutoGen 245 T using standard protocols. Genotyping was performed using TaqMan assays (rs616338, C___2270073_20; rs72824905, C__97909430_10; rs429358, C___3084793_20; rs7412, C____904973_10) following manufacturers protocol, using a QuantStudio 7 Flex Detection System with a 384-Well Block Module (Applied Biosystems, Foster City, CA).

### Sequencing

All minor allele carriers (*ABI3_*rs616338-T, *PLCG2*_rs72824905-G) were validated and confirmed using Sanger Sequencing. PCR primers with the following sequences were used to amplify and sequence the genomic region flanking the mutations: *ABI3* 5’-CTTCCTGCTCGCACCCGAC-3′, 5’-CTAATGCAGCATCCCCAACT-3′, *PLCG2* 5’-CCATAAATGAGGGCTCTCAG-3′, 5’-CATACCCACCTCACCCTTGT-3′. PCR products were purified using the Agencourt AMPure protocol (Beckman Coulter, CA) and sequenced using a Big Dye Terminator v3.1 Cycle Sequencing Kit (Applied Biosystems). Sequencing reactions were purified using Agencourt CleanSEQ (Beckman Coulter, CA) and run on an ABI33730xl Genetic Analyzer (Applied Biosystems). Sequences were analyzed using Sequencher 4.8 (Gene Codes Corporation, MI).

### Statistical analysis

The tests for association of *ABI3_*rs616338-T and *PLCG2*_rs72824905-G, with disease status, were implemented in PLINK [20] using logistic regression (LR) and Fisher’s exact test. An additive model for number of minor alleles with age, sex and *APOE* ε4 dose as covariates was used throughout, with series as an additional covariate for the AD association test in the Caucasian cohort. Subjects with incomplete age, sex and *APOE* genotype information were excluded. Each neurodegenerative disease cohort was tested separately for association with disease status using the same controls, with the exception of the African American AD cases who were compared to African American controls, and the AD Replication cohort which excluded the Mayo Clinic controls included in the Sims et al. [1] publication. Given that this study is pursuing replication of a previously reported association with AD, statistical significance was ascribed to *P* values < 0.05.

### RNA sequencing and analysis

RNA sequencing was performed on temporal cortex and cerebellum from 278 samples for which we already made all data available on the AMP-AD Knowledge Portal (synapse ID: syn5049298; syn3163039) [19]. Libraries were prepared using the TruSeq RNA Sample Prep Kit (Illumina, CA) according to manufacturer’s instructions and sequenced on an Illumina HiSeq 2000 at the Mayo Clinic Medical Genome Facility Gene Expression Core. Paired-end 100-bp raw reads were processed through MAP-Rseq bioinformatics pipeline [21] and were aligned to human genome build GRCh37 using TopHat aligner v2.0 [22]. Reads mapped to genes and exons were counted using featureCounts subroutine in Subread toolkit v1.4 [23], RSeQC v2.3.2 [24] and FastQC v0.10 (bioinformatics.babraham.ac.uk/projects/fastqc/) were applied to calculate quantity control (QC) measurements. A custom QC pipeline was applied to identify failed QC samples, which checked base-calling Phred score, number and percentage of reads mapped to genes, RNA degradation level, the consistency between estimated sex based on chromosome Y gene expression and recorded sex information, and sample distribution on the first two principle components. Samples which failed QC were excluded. Following QC, 86 AD, 84 PSP and 80 control samples with brain gene expression data were retained for analyses. Each of the remaining samples had a minimum of 64 million reads mapped to genes.

### Differential gene expression

Read counts of genes were normalized using R cqn package [25] which adjusted for library size, gene length and gene GC contents. For each gene, multiple linear regression was performed in which normalized gene expression was the dependent variable, diagnosis (AD vs. control; or PSP vs. control) is the independent variable of primary interest and sex, flowcell, age at death, RNA integrity number (RIN), and center from which the samples were obtained were the covariates (simple model). Additionally multiple linear regression was also run using the comprehensive model, which also included the expression values of five central nervous system (CNS) cell type markers as covariates in order to account for cell population variation in bulk brain tissue (neuron *ENO2*; endothelia *CD34*; microglia *CD68*; oligodendrocytes *OLIG2*; astrocytes *GFAP*), as previously established [26].

### Weighted gene co-expression network analysis (WGCNA)

To identify networks of genes co-expressed in the brain and their association with AD or PSP diagnosis, we applied WGCNA to our brain expression data as we previously reported [26]. Gene expression residuals were obtained from multiple linear regression using the simple model where gene expression was a dependent variable and sex, batch, age at death, RIN and center from which the samples were obtained were independent variables. Similarly, residuals from comprehensive model were obtained such that the expression of five cell marker genes were included as additional covariates as described above and previously [26]. WGCNA was performed on residuals obtained from the simple and comprehensive models independently, as described [26], using R WGCNA package [27]. WGCNA clusters co-expressed genes into modules such that genes in the same module have correlated expression levels. For each module, an eigengene is computed to represent the expression pattern of all genes in that module. Module eigengene is the first principal component of genes in the module and with sign adjusted so that it is positively correlated with mean expression of all genes. Module membership for a gene is defined as the Pearson correlation between that gene and eigengene of its module.

Co-expression networks were built using the pairwise diagnostic groups AD+Control and PSP + Control for temporal cortex and cerebellum gene expression levels separately to analyze associations of networks with diagnosis, as previously described [26]. For each pairwise diagnostic group, consensus modules were identified with the same softpower value and network type as for single group analysis using the “blockwiseConsensusModules” function. Pearson correlation is calculated between module eigengene and diagnosis to determine the association of the co-expression module with AD or PSP diagnosis. The modules are tested for (GO) enrichment using the “GOenrichmentAnalysis” function in WGCNA to ascribe biological processes to the co-expression modules. Network plots were generated for the WGCNA outputs using Cytoscape v3.2.0 (http://www.cytoscape.org/), as published [26].

### Co-expression network module cell type enrichment analysis

Co-expression network modules were annotated for presence and enrichment of genes that are primarily expressed in one of the five major cell types that exist in the CNS, i.e. neurons, oligodendrocytes, microglia, astrocytes and endothelia. Gene expression measures from these five sorted human brain cell populations were obtained from Zhang et al. [28] and analyzed as previously described [26]. Briefly, a gene was assigned one of the five CNS cell type if its mean expression value (in fragments per kilobase per million) in that cell type is > 4 times the mean expression in any other cell types. Using one-sided Fisher’s exact test, WGCNA modules were tested for enrichment of each of the five CNS cell types.

## Results

### Association of rs616338 and rs72824905 with AD

Genotypes were in Hardy-Weinberg equilibrium in all cohorts. In the Caucasian control subjects, we observed a minor allele frequency (MAF) of 0.010 for *ABI3_*rs616338-T and *PLCG2*_rs72824905-G (Table 2), closely emulating the reported gnomAD [29] frequencies in Non-Finnish Europeans of 0.00877 and 0.01 respectively. The AD risk association observed by Sims et al., with *ABI3_*rs616338-T was validated in our non-overlapping AD Replication cohort, which demonstrated an increased minor allele frequency (MAF) in the AD cases compared to the controls (MAF_AD_ = 0.016, MAF_controls_ = 0.011) despite the *P* values not reaching significance (Fisher’s odds ratio (OR) = 1.44, *p* = 0.116; LR OR = 1.49, *p* = 0.163). Consistent with the original report [1], *PLCG2*_rs72824905-G showed a decreased minor allele frequency in our AD Replication cohort compared to the controls (MAF_AD_ = 0.006, MAF_controls_ = 0.010), though this was not statistically significant (Fisher’s OR = 0.66, *p* = 0.192; LR OR = 0.86, *p* = 0.674). The complete Caucasian AD cohort showed significant association with both *ABI3_*rs616338-T (OR = 1.41, *p* = 0.044) and *PLCG2*_rs72824905-G (OR = 0.56, *p* = 0.008) with Fisher’s exact test. These OR estimates were maintained after multivariable logistic regression analyses, albeit at reduced significance (*ABI3*_rs616338-T OR = 1.36, *p* = 0.141; *PLCG2*_rs72824905-G OR = 0.58, *p* = 0.052).

**Table 2:** Association Results

a. *ABI3* rs616338													
Cohort		N		Genotype Counts (TT/CT/CC)		Minor Allele Frequency		Logistic Regression			Fisher’s		
		Cases	Controls	Cases	Controls	Cases	Controls	OR	P value	95% CI	OR	P value	95% CI
Caucasian	Replication AD	1479	1491	0/47/1432	0/33/1458	0.016	0.011	1.49	0.163	0.85–2.6	1.44	0.116	0.92–2.26
	AD	2742	3351	0/78/2664	0/68/3283	0.014	0.010	1.36	0.141	0.9–2.04	1.41	0.044	1.02–1.95
	PSP	231	3351	0/4/227	0/68/3283	0.009	0.010	0.86	0.773	0.3–2.42	0.85	1	0.31–2.35
	PD	837	3351	0/20/817	0/68/3283	0.012	0.010	1.62	0.140	0.85–3.07	1.18	0.504	0.71–1.95
	DLB	306	3351	0/11/295	0/68/3283	0.018	0.010	1.78	0.121	0.86–3.68	1.79	0.097	0.94–3.4
	MSA	128	3351	0/2/126	0/68/3283	0.008	0.010	0.76	0.716	0.17–3.41	0.77	1	0.19–3.15
African-American	AD	181	331	0/2/179	0/3/328	0.006	0.005	1.28	0.809	0.18–9.18	1.22	1	0.2–7.34
b. *PLCG2* rs72824905													
Cohort		N		Genotype Counts (GG/CG/CC)		Minor Allele Frequency		Logistic Regression			Fisher’s		
		Cases	Controls	Cases	Controls	Cases	Controls	OR	P value	95% CI	OR	P value	95% CI
Caucasain	Replication AD	1477	1487	0/19/1458	0/29/1458	0.006	0.010	0.86	0.674	0.41–1.77	0.66	0.192	0.37–1.18
	AD	2738	3346	0/31/270	0/67/3279	0.006	0.010	0.58	0.052	0.33–1.01	0.56	0.008	0.37–0.86
	PSP	231	3346	0/9/222	0/67/3279	0.019	0.010	1.86	0.099	0.89–3.91	1.97	0.061	0.97–3.97
	PD	853	3346	0/18/835	0/67/3279	0.011	0.010	1.28	0.511	0.62–2.63	1.05	0.788	0.62–1.78
	DLB	306	3346	0/2/304	0/67/3279	0.003	0.010	0.39	0.199	0.09–1.65	0.32	0.124	0.08–1.33
	MSA	127	3346	0/6/121	0/67/3279	0.024	0.010	2.71	0.059	0.96–7.62	2.39	0.050	1.03–5.57
African-American	AD	180	331	0/1/179	0/0/331	0.003	0.000	NA	NA	NA	NA	0.3523	NA

Results of Fisher’s exact test and multivariable logistic regression analysis, adjusted for age, sex and *APOE* ε4 dosage, testing for association of rs616338 and rs72824905 with disease status. In the Caucasian AD vs. control multivariable logistic regression analysis, series was also used as an additional covariate

*Abbreviations*: *OR* odds ratio, *CI* confidence interval

In the African-American control participants, *ABI3*_rs616338-T was very rare (MAF_AA-control_ = 0.005) and *PLCG2*_rs72824905-G was non-existent. Although the *ABI3* variant was observed more frequently in African-American AD cases than controls, this did not reach significance. Only one African-American AD case was a carrier of the *PLCG2* variant. The African-American control MAF that we observed for these variants were consistent with those reported in the Exome Variant Server [30] (*ABI3*_rs616338-*T* = 0.00368; *PLCG2*_rs72824905-G = 0.00079), which demonstrates lower frequencies compared with Caucasians, suggesting that these particular variants may have a lesser or no role for AD risk in African-Americans.

### Association of rs616338 and rs72824905 with other neurodegenerative diseases

**Fig. 1:**
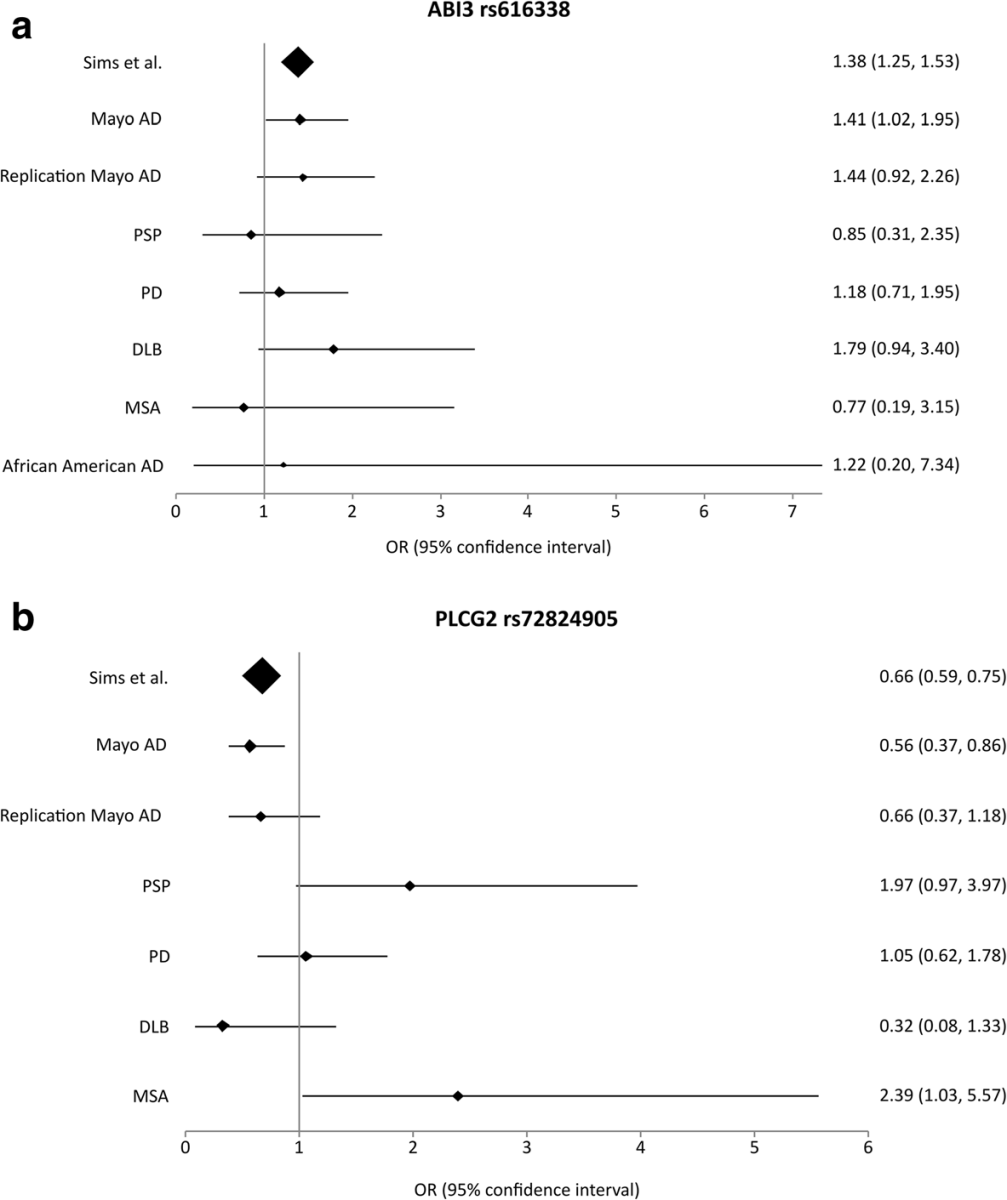
Forest plots of associations: Forest plots for Fisher’s exact test association results in each disease cohort with **a**. *ABI3* (rs616338) and **b**. *PLCG2* (rs72824905). ORs (diamonds) and 95% CI (whiskers) are plotted for each disease cohort and shown numerically on the right of each plot. Abbreviations: AD = Alzheimer’s disease; PSP = progressive supranuclear palsy; PD = Parkinson’s disease; DLB = dementia with Lewy bodies; MSA = multiple system atrophy

None of the other cohorts showed significant disease associations that were concordant with those for AD, although the DLB cohort had suggestive (0.05 < *P* < 0.2) findings with Fisher’s exact test (*ABI3*_rs616338-T OR = 1.79, *p* = 0.097; *PLCG2*_rs72824905-G OR = 0.32, *p* = 0.124) (Table 2; Fig. 1) and multivariable logistic regression analysis (*ABI3*_rs616338-T OR = 1.78, *p* = 0.121; *PLCG2*_rs72824905-G OR = 0.39, *p* = 0.199). The MAF of both variants was very similar between the DLB and AD patients in this study (MAF_DLB_ *ABI3*_rs616338-*T* = 0.018; *PLCG2*_rs72824905-G = 0.003), as is reflected in their overlapping OR estimates and 95% confidence intervals (CI) (Fig. 1).

In PD *ABI3*_rs616338-T showed a slightly higher MAF in cases compared to controls (MAF_PD_ = 0.012), however a suggestive association was observed only in the logistic regression analysis (LR OR = 1.62, *p* = 0.140; Fisher’s OR = 1.18, *p* = 0.504). No significant or suggestive association with PD was observed for *PLCG2*_rs72824905-G.

We observed a lower MAF for *ABI3*_rs616338-T in both PSP and MSA (MAF_PSP_ = 0.009, MAF_MSA_ = 0.007) compared to controls, which is converse to the findings in AD, although this did not reach statistical significance. Interestingly, *PLCG2*_rs72824905-G also showed evidence of association with both MSA and PSP in the opposite direction as that in AD (Table 2, Fig. 1). This variant had significant association with increased risk of MSA with Fisher’s test (OR = 2.39, *p* = 0.0499), and this association was maintained in the logistic regression analysis (OR = 2.71, *p* = 0.059). *PLCG2*_rs72824905-G also had suggestive association with increased risk of PSP (Fisher’s OR = 1.97, *p* = 0.061; LR OR = 1.86, *p* = 0.099). *PLCG2*_rs72824905-G MAF estimates for both MSA and PSP patients were about twice as much as that in controls (MAF_MSA_ = 0.024; MAF_PSP_ = 0.019).

A subset of the Caucasian controls were utilized in the prior publication [1] reporting *ABI3*_rs616338-T and *PLCG2*_rs72824905-G associations. We repeated all association tests with the other neurodegenerative diseases using the non-overlapping replication controls (*n* = 1491), in the unlikely event that the original control cohort might harbor any potential bias that might influence these results. As expected, the findings from the association tests using the replication controls (Additional file 1: Table S1) were very similar to those obtained from the full set of controls.

### Brain expression of *ABI3* and *PLCG2*

Using the Mayo Clinic RNAseq data [19], we previously showed that both *ABI3* and *PLCG2* had higher expression levels in the temporal cortex of AD brains compared to controls, although the significance was abolished upon adjusting for CNS cell type markers [1]. In this study, we also examined differential expression of these genes in PSP vs. control brains; and evaluated an additional brain region, cerebellum, which unlike temporal cortex, is relatively spared from AD neuropathology. We determined that none of the other analyses revealed significant differential expression for either *ABI3* or *PLCG2* (Additional file 1: Table S2). Specifically, there was no significant differential expression of these genes in the cerebellum for either AD or PSP against controls, using either the simple model, which does not adjust for cell type markers; or the comprehensive model, which does. In the temporal cortex, significant differential expression of *ABI3* (ß = 0.58, *p* = 1.99E-03) and *PLCG2* (ß = 0.40, *p* = 7.37E-03) was observed only for the AD vs. control analysis under the simple model but not the comprehensive model, as we previously reported [1].

To determine the network of co-expressed genes within which *ABI3* and/or *PLCG2* reside, we performed WGCNA [27] of genes expressed above a pre-determined threshold (median CQN value> 0) within the temporal cortex or cerebellum using the simple model. Both genes met the threshold requirements for the cerebellum analysis, however *PLCG2* was excluded from the temporal cortex WGCNA due to its low median expression in this region. In the cerebellum, *ABI3* and *PLCG2* were members of the same co-expression network modules identified in the AD+Control and PSP + Control brains. These networks were highly enriched for microglial genes as well as the “immune response” GO term (Additional file 1: Table S3). In the temporal cortex, *ABI3* also belonged in the “immune/microglial” modules, which harbored many other AD candidate risk genes [10, 31] (Additional file 1: Table S4, Additional file 2: Figure S1).

We determined that the immune/microglial module had significantly higher expression in the temporal cortex of AD samples compared to controls (ß = 0.21, *p* = 9.55E-03) (Additional file 1: Table S3). In this module, named AD+Ctrl.TCX14, *ABI3* is one of the most well-connected genes with a module membership (MM) of 0.90, which places it as the fourth most well-connected AD candidate risk gene behind *TYROBP, SPI1*, and *MS4A6A* (Additional file 1: Table S4) and the 35th of 246 genes in this module (data not shown). The cerebellar immune/microglial module did not have significant association with AD, unlike that of the temporal cortex. Neither the cerebellar nor the temporal cortex immune/microglial modules showed any significantly different expression in the PSP vs. control brains (Additional file 1: Table S3). All three immune/microglial modules, which lacked disease association harbored many of the genes from the AD+Ctrl.TCX14 module, including *ABI3*, which has high MM (0.82–0.92) in all three modules, and *PLCG2* in the two cerebellar modules (MM = 0.69–0.71). This suggests that the lack of association is unlikely to be due to differences in the structure of co-expression, but rather due to the different levels of these genes in the AD temporal cortex.

Under the comprehensive model adjusting for CNS cell type markers, there was no longer an immune/microglial module in the temporal cortex with significant AD association. This is likely due to the fact that *CD68*, which was used as the microglial marker for cell type adjustments in the comprehensive model, is a gene with strong correlations within AD+Ctrl.TCX14. This is evident from its high MM of 0.96 (Additional file 1: Table S4, Additional file 2: Figure S1), making it the 4th most well-connected gene in this module, where *TYROBP* is the top most-connected gene. Hence, inclusion of *CD68* levels in the analytic model, as a cell type marker, abolishes the transcriptional correlations within and AD association of the temporal cortex immune/microglial network, suggesting that this association is likely driven by either microgliosis, microglial activation or both.

## Discussion

In this study we evaluated the association of the recently discovered [1] variants in the microglia-enriched genes *ABI3* and *PLCG2* in AD and four other neurodegenerative diseases comprised of three α-synucleinopathies (PD, DLB, MSA) and a primary tauopathy (PSP); investigated an African-American AD case-control cohort for presence and frequency of these variants; and studied the expression patterns of these genes in two brain regions (temporal cortex, cerebellum) in AD vs. control; and PSP vs. control samples. We validated the associations previously reported with *ABI3_*rs616338-T (p.Ser209Phe) and *PLCG2*_rs72824905-G (p.Pro522Arg) in a Caucasian AD case-control cohort, and observed a similar direction of effect in DLB. In contrast, the effect estimates observed for PSP and MSA were in the opposite direction to that in AD for both variants. Neither of the two variants appeared to associate with risk of PD and they both had exceedingly rare frequencies in the African-American AD case-control cohort.

Similar direction of effect for both variants in AD and DLB, despite lack of or opposite trends of association in other α-synucleinopathies (PD, MSA) or a tauopathy (PSP) may be due to high rates of existing AD pathology in DLB, observed in about 2/3 of autopsy-proven DLB patients [32, 33]. It is also possible that these variants represent a shared genetic component between AD and DLB, similar to *APOE* [34–36], the effects of which have also been demonstrated in pure DLB [36]. AD and DLB share similarities with respect to neuroinflammation, such as microglial activation and cytokine induction, which are observed in both neuropathology studies of these diseases, as well as in vitro studies of effects of Aß or α-synuclein on microglia [33]. Thus, it remains a possibility that the potential effects of *ABI3_*rs616338-T and *PLCG2*_rs72824905-G on microglial function may influence AD and DLB risk, similarly. It should be noted that in a recent study of a larger DLB cohort, including 829 pathologic Lewy body diseases (LBD) patients, another microglial gene variant *TREM2* p.R47H was found to associate with disease risk only in those LBD patients with predominant AD pathology, but not in those with low AD pathology [37], indicating lack of a role for this variant in DLB pathophysiology. In our study, only 67 of 306 DLB patients were autopsy confirmed, hence we were not powered to test for genetic associations while adjusting for the presence of concomitant AD pathology. Future studies of pathologically confirmed DLB cohorts with low or no AD pathology are needed to distinguish the effects of *ABI3_*rs616338-T and *PLCG2*_rs72824905-G on pure DLB.

One surprising finding from our study is the identification of opposite trends of association for these variants with both PSP and MSA patients, which reached nominal significance for *PLCG2*_rs72824905-G in the MSA cohort but were not statistically significant in the other comparisons. These results could merely represent false positive findings given the rarity of the tested variants and the relatively small PSP and MSA cohorts, although all patients for both diseases were autopsy proven, which ensures diagnostic accuracy. Nevertheless, despite its rarity, the higher MAF of *PLCG2*_rs72824905-G in PSP and MSA which is ~ 2–2.4 times as that of controls and 3–4 times as that of AD patients, raises the possibility of opposite effects of this variant in AD vs. these two neurodegenerative diseases. To hypothesize on the biological basis of such opposing effects, the potential effect of the *PLCG2*_rs72824905-G variant on immune function needs to be considered.

*PLCG2*_rs72824905-G causes a proline to arginine substitution (p.Pro522Arg), located between the nspPH and nSH2 domains, which comprise part of the autoinhibitory region of PLCγ2 [38]. In vitro studies have demonstrated that deletion of amino acids in the nspPH-nSH2 linker domain cause an increase in PLCγ2 activity, both basal and in response to stimulation [38]. Deletions [8] and a missense [9] variant within the nSH2 domain in humans have been shown to cause PLAID and APLAID (p.Ser707Tyr), respectively [39]. These diseases are characterized by immune dysregulation, antibody deficiency and autoinflammation due to a complex mix of loss and gain of function of PLCγ2 [39]. Additionally, point mutations in the nspPH domain in a murine model have also been shown to increase PLCγ2 activity [40] and in vivo studies have demonstrated that *PLCG2* gain of function mutations can lead to autoinflammation [7]. The *PLCG2*_rs72824905-G variant causes an amino change from a non-charged to a positively charged residue, likely altering the structure of the protein. This can affect the interaction of the autoinhibitory and catalytic regions, consequently reducing the autoinhibition of PLCγ2. Thus, *PLCG2*_rs72824905-G is expected to increase the PLCγ2 signaling activity, which may induce activation of inflammation and innate immunity [5, 6].

Identification of AD candidate genes and risk variants in innate immunity pathways [41], enriched expression of many of these genes in microglia [28], gene expression network [42] and expression quantitative trait loci (eQTL) studies [43–46] implicating regulatory changes of these genes and variants in brain tissue collectively provide strong evidence for role of innate immunity in AD. Rare, coding *TREM2* variants that increase risk of AD appear to have loss of function effects and are associated with reduced amyloid plaque-associated microgliosis [47]. A common potentially regulatory variant that is associated with modestly higher brain levels of *TREM2*, also associates with a protective effect in AD [46]. Consistent with this, elevated *TREM2* gene dosage reduced amyloid pathology and improved memory in a mouse model of AD [48]. These studies suggest that enhanced function or increased levels of microglial, innate immunity genes may confer protection in AD, especially through their effects on Aß clearance.

In contrast, enhanced innate immunity may have detrimental effects in non-AD neurodegenerative diseases or in non-Aß components of AD pathology. Complement and microglial activation increased tau pathology in mouse models, whereas their inhibition and depletion, respectively, reduced synapse/neuron loss and tau pathology (reviewed [10]). Microglia were found to induce neuron-to-neuron spread of tau in an adeno-associated virus–based mouse model [49]. Although prion-like spread has been suspected for tau in PSP and α-synuclein in MSA [50], the role of microglia in propagation of proteinopathy in these diseases has not been demonstrated. Neuroinflammation and microglial activation is observed in both PSP [51, 52] and MSA [53] in disease affected brain regions, although whether this is beneficial or detrimental to the disease progress remains to be established.

In light of these collective data, one model, which may reconcile the opposing trends in the effects of *PLCG2* and *ABI3* variants in AD vs. PSP and MSA in our study is that activation of innate immunity relatively early in the neurodegenerative process may be beneficial, especially in the context of extracellular Aß pathology. However, persistently activated innate immunity and microglia may be detrimental for propagation of intracellular tau or α-synuclein, which characterizes diseases such as PSP and MSA, as well as for late-stage neurodegeneration. It should be emphasized that the genetic association findings in our study are statistically marginal, and until replicated in other cohorts, the above model remains speculative.

Our study also investigated an African-American AD case-control cohort for associations with *ABI3_*rs616338-T and *PLCG2*_rs72824905-G. While this cohort is of modest size, it enabled the observation that MAF for both variants are smaller than those of both Caucasians AD and elderly control patients. This suggests that these variants are unlikely to influence AD risk in African-Americans to the extent observed in Caucasians. This does not, however, rule out *ABI3* or *PLCG2* as potential AD risk genes in African-Americans. We have identified *TREM2* coding variants that confer AD risk in African-Americans [54], but these were different than the AD risk variants identified in Caucasian subjects [55, 56]. Deep sequencing efforts on African-Americans and other non-Caucasian races are necessary to identify the full spectrum of AD risk variants in these and other genes.

We also characterized the gene expression patterns and co-expression networks of *ABI3* and *PLCG2* in two brain regions, namely temporal cortex that is affected with AD neuropathology and cerebellum that is relatively spared, in AD, PSP and control samples. PSP was included in the expression analysis as a neurodegenerative disease, which like AD, has tau pathology, but unlike AD, lacks Aß pathology, and as such may help distinguish expression changes in the context of these different neuropathologies. We previously showed that comparative transcriptomics utilizing different neurodegenerative diseases may identify pathways that are commonly vs. distinctively perturbed in these conditions [26]. We determined that temporal cortex, but not cerebellum, levels of these genes and their co-expression networks are increased in AD, but not in PSP, compared to control samples. These elevations are abolished after adjusting for levels of cell type markers, including CD68, which is highly expressed by monocytes and macrophages and upregulated in actively phagocytic cells [57]. Our findings suggest that *ABI3* and *PLCG2* are members of a network of co-expressed microglial genes, the levels of which are upregulated in brain regions affected by AD pathology, where this upregulation is either the result of enhanced numbers or activation state of microglia or both. In this study, we utilized our gene expression data from the AMP-AD Consortium [19], which is primarily focused on brain regions affected with or relatively spared in AD. The fact that we did not observe microglial transcript elevations in PSP suggests that either such microgliosis is not a key aspect of this disease or that PSP-affected brain regions should be examined to observe such changes. Future studies focusing on brain regions affected in PSP are needed to address these possibilities. Although the gene expression changes we observed in AD may be driven by the microglial response to AD pathology, the presence of many AD candidate risk genes in these immune/microglial networks suggests that perturbed function or levels of these genes and networks are likely to play a causal role in AD pathophysiology.

In summary, our study provides effect size estimates for the recently discovered *ABI3* and *PLCG2* variants [1] in five different neurodegenerative diseases and an African-American AD case-control cohort and also characterizes the expression patterns of these genes in two brain regions in AD, PSP and controls. The strengths of our study are the sizable AD case-control cohort, evaluation of a variety of neurodegenerative diseases and an African-American cohort, neuropathologic diagnosis of all PSP and MSA and > 40% of AD cases and thorough examination of gene and co-expression networks for these genes. Despite these strengths, the sizes for the non-AD neurodegenerative cohorts remain modest, therefore assessment of larger cohorts for replication of the findings is necessary.

## Conclusions

Our findings strengthen the evidence for the role of *ABI3_*rs616338-T and *PLCG2*_rs72824905-G as risk and protective factors, respectively, for AD and suggest a similar role for these variants in DLB, but suggest opposite effects, especially for *PLCG2*_rs72824905-G in PSP and MSA. These and our gene expression data provide a rationale to investigate the roles of these and other innate immunity genes for their distinct roles in different neurodegenerative diseases.

## Additional files

Additional file 1: **Table S1.** Association Results with the Replication Controls. **Table S2.** Differential Gene Expression. **Table S3.** Microglial gene enriched co-expression network modules. **Table S4.** AD candidate risk genes in the microglial/innate immune module. (XLSX 28 kb)

Additional file 2: **Figure S1.** Network plot for the microglial/innate immune module: AD+Ctrl.TCX14, based on the temporal cortex expression levels from AD and control samples, under the simple model. The top 150 connections according to topological overlap matrices weight from WGCNA are shown for genes with a module membership (MM) > 0.7. The size of the node correlates to the number of connections of that node with others in the network, and green denotes genes enriched in microglia. Transcripts with significant differential expression between AD and controls with q < 0.05 are shown as a square. (TIFF 6088 kb)

## Abbreviations

ABI3: Abelson (Abl) interactor (Abi) family protein 3 (a.k.a. NESH)
AD: Alzheimer’s disease
APLAID: Autoinflammation and PLCɣ2-associated antibody deficiency (PLAID)
Arp2/3: Actin-related protein-2/3
Aβ: Amyloid beta
Ca^2+^: Calcium
CER: Cerebellum
CNS: Central nervous system
DAG: Diacylglycerol
DLB: Dementia with Lewy bodies
eQTL: Expression quantitative trait loci
ERK: Extracellular signal-regulated kinase
GO: Gene ontology
GS: Gene significance
HSPC300: Hematopoietic stem progenitor cell 300
IP2: Inositol 1,4,5-trisphosphate
LBD: Lewy body diseases
MAF: Minor allele frequency
MM: Module membership
MSA: Multiple system atrophy
NAP: NCK-associated proteins
PD: Parkinson’s disease
PIP2: Phosphatidylinositol 4,5-bisphosphate
PLAID: PLCɣ2-associated antibody deficiency
PLCG2: Phospholipase C family protein (PLCɣ2)
PSP: Progressive supranuclear palsy
RIN: RNA integrity number
SRA1: Specifically Rac-associated 1
TCX: Temporal cortex
WAVE2: WASp-family verprolin homologous protein 2
WGCNA: Weighted Gene Co-expression Network Analysis
