# Supplementary figures and images for: ABI3 and PLCG2 missense variants as risk factors for neurodegenerative diseases in Caucasians and African Americans
Source: Mol Neurodegener. 2018 Oct 11;13:53. doi: 10.1186/s13024-018-0289-x (PMC6190665; doi:10.1186/s13024-018-0289-x)

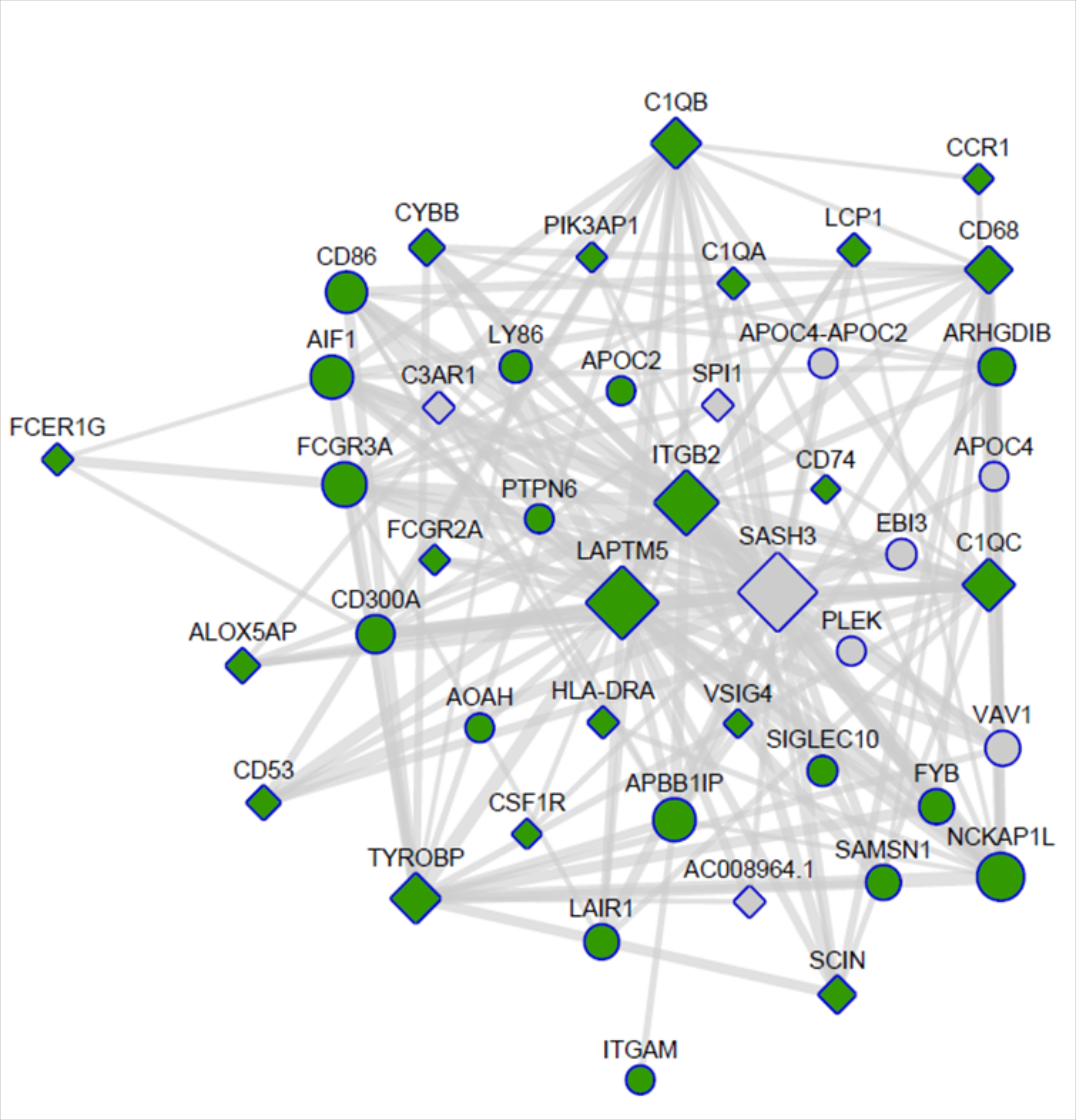

Supplement: Supplementary file 2 — Figure S1. Network plot for the microglial/innate immune module: AD+Ctrl.TCX14, based on the temporal cortex expression levels from AD and control samples, under the simple model. The top 150 connections according to topological overlap matrices weight from WGCNA are shown for genes with a module membership (MM) > 0.7. The size of the node correlates to the number of connections of that node with others in the network, and green denotes genes enriched in microglia. Transcripts with significant differential expression between AD and controls with q < 0.05 are shown as a square. (TIFF 6088 kb) [file 13024_2018_289_MOESM2_ESM.tiff]
